# Equitable and culturally sensitive perinatal mental health screening and referral for all: experiences and needs from primary care and community-based healthcare providers

**DOI:** 10.1186/s12913-026-14346-z

**Published:** 2026-03-11

**Authors:** Floor Puttemans, Titia Hompes, Leontien Jansen, Vasiliki Andreou, Ronny Bruffaerts, Lore Lannoo, Patrick Luyten, Anne Smits, Kristel Van Calsteren, Birgitte Schoenmakers

**Affiliations:** 1 Department of Neurosciences, Mind-body Research, KU Leuven, Leuven, Belgium; 2 Department of Public Health and Primary Care, Academic Center for General Practice, KU Leuven, Leuven, Belgium; 3 Adult Psychiatry, University Psychiatric Center KU Leuven, Leuven, Belgium; 4 Department of Neurosciences, Public Health Psychiatry, KU Leuven, Leuven, Belgium; 5 Institute of Medical Education Research Rotterdam, Erasmus MC, Rotterdam, The Netherlands; 6 Department of Development and Regeneration, Woman and Child, KU Leuven, Leuven, Belgium; 7 Department of Obstetrics & Gynaecology, Feto-maternal medicine, University Hospitals Leuven, Leuven, Belgium; 8 Faculty of Psychology and Educational Sciences, Clinical Psychology, KU Leuven, Leuven, Belgium; 9 Research Department of Clinical, Educational and Health Psychology, University College London, London, UK; 10 Neonatal Intensive Care Unit, University Hospitals Leuven, Leuven, Belgium

**Keywords:** Perinatal well-being, Postnatal depression, Detection, Risk assessment, Perspectives, Barriers, Facilitators, Migration, Clinicians, Qualitative research

## Abstract

**Background:**

Perinatal mental health (PMH) problems are common, in particular among perinatal women from migrant backgrounds. Despite global recommendations for universal screening, implementation remains inconsistent and inequitable. To better understand these inequities, this study aimed to explore healthcare providers’ experiences and needs regarding screening and referral for perinatal depression and anxiety among women from migrant backgrounds in Flanders, Belgium.

**Methods:**

A qualitative study design was employed, conducting focus groups with primary care and community-based healthcare providers. Participants were recruited through a purposive sampling strategy using newsletters, social media, and email. Discussions were recorded, verbatim transcribed, and analysed using inductive framework analysis. To ensure credibility, a member check survey was conducted.

**Results:**

Based on the analysis of five heterogeneous focus groups (*n* = 20 in total), four themes and seven subthemes were identified: (1) complexity of interactions between healthcare providers and migrant women: underscoring (a) the impact of cultural and language barriers and (b) the importance of a trusting relationship; (2) screening in its context: highlighting (c) the ambivalence around screening and (d) the impact of organisational and financial factors on implementation; (3) accessible referral: stressing (e) the importance of person-centred, culturally and linguistically appropriate services; and (4) PMH in its proper context: addressing (f) the role of stigma, taboo, PMH literacy, and understanding, and (g) the involvement of women’s partner, family and broader network.

**Conclusions:**

These findings underscore the complexity of screening and referral practices among all (expectant) mothers, particularly among women from migrant communities, and simultaneously offer a foundation to develop and implement screening and referral protocols. Ideally, these protocols should be co-designed with perinatal service users to ensure equitable practices for all.

**Supplementary Information:**

The online version contains supplementary material available at 10.1186/s12913-026-14346-z.

## Background

The perinatal period, defined as pregnancy until one year postpartum, is a critical phase full of change, associated with a heightened risk for (re)experiencing mental health problems [1]. Affecting 12–17% and 15% of (expectant) mothers respectively, perinatal depression and anxiety are the most common perinatal mental health (PMH) problems [2–4]. PMH problems do not only affect (expectant) mothers [5], their children [6] and their partners [7–9], but also create long-term societal costs [10, 11], making it a critical concern to public health. To minimise its impact and provide timely support, identification of signs of PMH problems is crucial [12, 13]. Routine screening has been globally recommended [14], and can be performed using various self-report instruments [15]. Screening is typically conducted by healthcare providers (HCPs) in primary or tertiary care settings [15]. Alternatively, community-based screening offers an accessible option for reaching underserved perinatal women [16]. Positive screening results should lead to referral to appropriate services, including mental health care [15].

With increased global migration, there is a growing number of (expectant) mothers having children outside their country of origin [17]. Not only are they a substantial group, but they also face a heightened risk of PMH problems [18]. Specifically, among women from first-generation migrant backgrounds, the pooled prevalence of perinatal depression is estimated at 24.2%, and perinatal anxiety affects 19.6%. These estimates are based on both diagnosed cases and positive screening outcomes reported across multiple studies included in a recent systematic review and meta-analysis, and represent rates considerably higher than those observed in mothers with a non-migrant background [18]. Given this heightened prevalence, PMH screening and referral among migrant communities are crucial. However, research indicates that current practices are inequitable, with a greater risk of lacking screening or referral among women from migrant backgrounds [19–24]. For example, in a recent Danish study, migrant women were 81–90% more likely to miss postpartum depression screening, compared to non-migrant women [20]. These disparities may be due to women declining screening or referral for various reasons, such as different understandings of PMH, prior negative healthcare experiences, fear of consequences for their child, and concerns about shame for their partner or family [24–26]. Also, HCPs may be less inclined to offer screening to this group due to challenges in clinical practice, such as managing cultural and linguistic differences [27]. Despite using translated screening instruments or interpreters, many HCPs feel frustrated and report difficulties in recognising depression and managing cultural differences, expressing a limited belief in their ability to perform these tasks [27, 28]. Additionally, HCPs might be less likely to refer women, due to various reasons such as time constraints [26], lack of knowledge or available services [24], or challenges in motivating women to seek external help [29]. When women refuse to engage with external care, HCPs often step in to provide counselling themselves, which may lead to women not receiving specialised care [29].

Despite growing recognition of the challenges faced by HCPs in supporting women from diverse backgrounds, research so far has mainly focused on first-generation migrant groups [26]. This narrow focus overlooks the broader and increasingly relevant issues related to cultural and linguistic diversity among later generations and minority populations. Expanding the focus beyond first-generation migrant women could offer more inclusive insights into structural barriers that affect access to and quality of perinatal mental healthcare, regardless of their specific migration status or origin. Moreover, little is known about the challenges HCPs face during the antenatal period, a critical window in which early screening and referral are essential to prevent the progression of PMH problems into the postpartum period. Understanding how HCPs experience practices during pregnancy is therefore important to explore. Additionally, community-based settings may better reach women from migrant backgrounds due to their accessibility, yet insight into HCPs’ perspectives of screening in these contexts remains limited [16]. Finally, prior studies have mainly been focused on countries with relatively well-integrated healthcare systems for perinatal care, such as in the United Kingdom and the United States of America, leaving a considerable gap in our understanding of HCPs’ experiences in other healthcare systems, where standardised protocols are lacking or are inconsistently applied [30]. A deeper exploration of HCPs’ experiences and needs is therefore needed to understand how individual, interpersonal and system-level factors influence their ability to effectively screen and refer for PMH problems.

To this end, the present study aims to explore HCPs’ experiences related to screening and referral of perinatal depression and anxiety among women from migrant backgrounds in primary care and community-based settings in Flanders, Belgium. The study was guided by the following research questions: (1) how do HCPs screen and refer for PMH problems among women from migrant backgrounds? (2) how do they experience this process and what are their views on screening and referral? (3) which barriers and facilitators impact screening and referral behaviour? and (4) what are their needs to screen and refer in a culturally sensitive way?

## Methods

### Study design

To address our research questions, we conducted a generic qualitative study using focus groups with HCPs [31]. Data were analysed based on an inductive approach, i.e. the Framework Method [32]. The study was reported according to the Consolidated Criteria for Reporting Qualitative Research (COREQ) [33].

### Setting

This study is part of a larger research project, i.e. the Perinatal mental health Risk profiling and Intervention in Leuven study (PRIL-study). Considering the unique reality of working with women from migrant backgrounds, focus groups were organised to better understand how PMH screening and referral are experienced in primary care and community-based settings by HCPs. The focus groups were organised in two different settings, i.e. in the University Hospitals of Leuven and in a primary care group practice, outside the hospital.

### Participants

We employed maximum variation purposive sampling for recruitment based on two predefined criteria, i.e. professional role and organisational setting, which were selected to ensure a broad range of perspectives across different functions and contexts relevant to the study. We recruited HCPs working in primary care and community-based settings in the wide region of the medium-sized city of Leuven, Belgium. HCPs were included if they were [1] involved in the perinatal care for and support of women from migrant backgrounds, and [2] Dutch-speaking. HCPs of interest were GPs, midwives, maternity nurses, those working for child health services, gynaecologists, physiotherapists, primary care psychologists, and cultural support workers. Cultural support workers, also called ‘cultural brokers’ or ‘intercultural mediators’, were bilingual/bicultural interpreters, helping to bridge cultural gaps and facilitate understanding in healthcare and social welfare. Recruitment was organised in several phases. Prior to the actual start of the study, a research team member (FP) contacted the following healthcare organisations to identify their potential interest in study participation: Flemish and regional professional associations for GPs (n = 2), for midwives (n = 2), and for physiotherapists (n = 1), social care organisations (n = 4), Flemish and regional child health services (n = 5), local child and family support community centres (n = 16), local general community centres (n = 9), local community health centres (n = 3), regional primary care zones (n = 3), regional mental health networks (n = 3), and multi- and monodisciplinary private practices for GPs, midwives and physiotherapists (n = 45). Information about the study was subsequently disseminated via newsletters of those organisations, social media, or email. Interested individuals could contact FP directly, after which they received study details, and a suitable focus group time was scheduled.

### Data collection

Focus groups were organised between November 2024 and January 2025. We aimed for three to five focus groups [34], each consisting of four to eight participants [35], to ensure a diversity of perspectives, while nurturing a comfortable environment for sharing personal experiences [36]. Groups were composed heterogeneously in terms of professional background, to mirror clinical practice, stimulate discussion, and limit group thinking [37]. Final group composition was guided by participant, researcher, and location availability. A topic guide was developed based on existing literature and team discussions (Additional file 1). To test the guide, it was piloted with six HCPs from the Gynaecology and Obstetrics ward at the University Hospitals of Leuven (see "Setting"). Data from this pilot served solely to refine the guide and were executed from the analysis. After each focus group, the topic guide was further refined to deepen our understanding of the derived themes. Focus groups were held in Dutch. A moderator (FP) guided the discussions, while an observer (EC for focus group 1 and 4 and LM for focus group 2, 3 and 5) provided practical support and made field notes on group dynamics and non-verbal communication. Each session started with a short introduction by the moderator and observer, an explanation of the study and the discussion topic, and brief definitions of key concepts. Participants then introduced themselves, before engaging in a structured discussion on the following pre-defined topics: [1] experience with working with mothers from a migrant background; [2] experience with talking about PMH with them; [3] experience with PMH screening and referral among them: current practice, barriers, facilitators; [4] perspectives on future screening and referral: needs regarding approach and setting, and perspectives on strategies to facilitate implementation in clinical practice. All sessions were audio- and video-recorded. Data collection continued until data saturation was achieved, i.e. the point at which no new information emerged [36].

### Data analysis

Data were analysed following the Framework Method with an inductive approach [32]. All seven stages described by Gale et al. (2013) were completed [32]. First, focus groups were pseudonymized and transcribed verbatim with the assistance of an online AI transcription service [38]. Second, two researchers (FP and VA) independently read the first transcript, making notes of thoughts and impressions while reading. Third, they independently identified relevant themes and concepts. In a fourth stage, they compared their coding and agreed on an initial set of codes, which FP used to create a working analytic framework, structured around the research questions, and refined in consultation with the research team. At the fifth stage, FP indexed the subsequent transcripts in the framework, adapting it to ensure alignment with the data. During this process, overlapping themes were identified, leading to a further adaptation of the framework. Data was charted into a framework matrix, which at the final stage was discussed and interpreted by the whole research team. NVivo (version 15) [39] was used to facilitate the process.

### Member check

To enhance the credibility of our results, we conducted a member check by sending a short survey to participants (Additional file 2) [40]. Via email, we shared an infographic summarising the results. We asked participants to approve, reject, or suggest modifications to the infographic by providing their impressions, indicating their level of recognition, and suggesting any necessary adjustments.

### Reflexivity statement

We acknowledge that our professional and personal experiences shaped the research process. To ensure rigour, the first author kept a reflexivity diary, the observers made field notes during each focus group, and post-session debriefings were kept reflecting on group dynamics and key discussion points. Our research team brought together diverse disciplinary backgrounds and perspectives: a female PhD student with a background in physiotherapy in mental health care (FP), two female postdoctoral researchers – one with a background in educational sciences (VA) and one in public health psychiatry (LJ), a male professor in Psychology and clinical psychologist (PL), and six professors in Medicine – one female perinatal psychiatrist (TH), one female neonatologist (AS), two female gynaecologists (LL, KVC), one male clinical psychologist (RB) and one female GP (BS). Data analysis was conducted by FP and VA, both trained in qualitative research, with VA having over five years of experience. Preliminary findings were discussed with the full team, including two senior qualitative researchers (LJ, BS).

## Results

### Participants

Five focus groups were conducted, averaging 81 min each, with three to five HCPs per group, totalling twenty participants. All but one group were heterogeneous in terms of professional, organisational and migrant backgrounds. Five HCPs were unable to attend due to illness, scheduling issues, and unspecified reasons. Participants’ characteristics and group composition are detailed in Table 1.

**Table 1:** Participants’ characteristics and focus group compositions

		Focus group 1 (*n* = 4)	Focus group 2 (*n* = 6)	Focus group 3 (*n* = 3)	Focus group 4 (*n* = 3)	Focus group 5 (*n* = 4)
Age (years) (mean; SD)		33; 2	38; 9	42; 5	50; 17	39; 6
Profession		• Midwife (*n* = 1) • Maternity nurse (*n* = 2) • Nurse (*n* = 1)	• GP (*n* = 1) • Maternity nurse coordinator (*n* = 2) • Cultural support worker (*n* = 1) • Family support worker (*n* = 1) • Social worker (*n* = 1)	• GP (*n* = 1) • Community worker (*n* = 1) • Psycho-pedagogue (*n* = 1)	• Midwife (*n* = 3)	• GP (*n* = 1) • Physiotherapist (*n* = 1) • Primary care psychologist (*n* = 1) • Obstetrician (*n* = 1)
Organisational setting		• Social care organisation (*n* = 3) • Child health services (*n* = 1)	• Community health centre (*n* = 1) • Social care organisation (*n* = 2) • Community centre (*n* = 1) • Child health services (*n* = 2)	• Private GP practice (*n* = 1) • Child and family support community centre (*n* = 1) • Child health services (*n* = 1)	• Private midwifery practice (*n* = 2) • Community health centre (*n* = 1)	• Community health centre (*n* = 1) • Private physiotherapy practice (*n* = 1) • Community mental health centre (*n* = 1) • Regional general hospital (*n* = 1)
Migrant status	Country of birth	• Belgium (*n* = 3) • Germany (*n* = 1)	• Belgium (*n* = 4) • Syria (*n* = 2)	• Belgium (*n* = 2) • Somalia (*n* = 1)	• Belgium (*n* = 3)	• Belgium (*n* = 3) • Australia (*n* = 1)
	Language spoken at home	• Dutch (*n* = 4)	• Dutch (*n* = 4), • Arabic (*n* = 2)	• Dutch (*n* = 2) • Somali (*n* = 1)	• Dutch (*n* = 3)	• Dutch (*n* = 3) • English (*n* = 1)

Abbreviations: GP, General practitioner

### Main findings

Four overarching themes and seven subthemes were derived from the analysis: (1) complexity of interactions; (2) screening in its context; (3) accessible referral; and (4) PMH in its proper context (Table 2). In the following section, the themes and subthemes are further illuminated with quotes which were translated from Dutch to English and shortened and edited to improve clarity and readability [41].

**Table 2:** Themes and subthemes

Theme	Subtheme
Complexity of interactions	Cultural and language barriers
	Trusting relationship
Screening in its context	Ambivalence around screening
	Organisational and financial factors
Accessible referral	Accessible referral
PMH in its proper context	Stigma, taboo, PMH literacy and understanding of PMH
	Involvement of ‘the village’

Abbreviations: PMH; Perinatal mental health

#### Complexity of interactions

##### Cultural and language barriers

Differences in cultural background or language were frequently mentioned as challenging, requiring more time, and sometimes evoking feelings of irritation, discomfort and a sense of failure. When cultural differences emerged, including in (non-)verbal communication styles, emotional expression, lifestyle and upbringing, HCPs struggled to interpret these differences. Cultural support workers were seen as valuable support through interpreting and helping navigate these cultural differences. Language barriers were not limited to the absence of a shared language; even when using common languages such as English or French, HCPs voiced difficulties regarding nuanced mental health discussions. HCPs used various strategies, including body language, visual aids, translation apps, simplified speech or interpreters. However, these efforts seemed to be hindered by practical barriers such as service availability or costs, limited knowledge of available support, or concerns about the perceived impersonal nature or reliability of tools.

*But if they don’t speak the language*,* we have a list with translations*,* and I find it very difficult to just turn the computer around and show that translation in order to get a truly honest answer. It feels very artificial*,* and for parents*,* it also comes across quite strongly. [FG1*,* nurse working for child health services]*

Many valued interpreting services, yet others questioned their effectiveness or need. Interactions were currently facilitated by interpreting services offering diverse languages and immediate availability, as well as audio-enabled translation apps; however, HCPs emphasised the continued need for accessible tools such as illustrations or online informative platforms. Nonetheless, cultural and language differences did not hinder all HCPs, as illustrated by the following quote.

*We may be different in language; we may be different in culture. But in my experience as a caregiver*,* if you have a good communicative foundation and can build trust*,* just by making that humane connection with someone*,* then language or culture in itself doesn’t necessarily have to be an obstacle. You can still build an equally meaningful relationship with someone*,* despite the difference in language and culture. [FG2*,* GP working in a community health centre]*

##### Trusting relationship

A trusting relationship was understood as essential for disclosure and (acceptance of) referral, with additional layers arising when working with women from migrant backgrounds. HCPs noted that some women preferred culturally similar HCPs and occasionally refused care from unfamiliar professionals, which raised concerns about one’s professional role or boundaries.

*It’s tough*,* but sometimes I think ‘gosh it seems like I’m always working’. If I’m taking my little boy to school*,* the whole day*,* people calling*,* stopping me… [FG2*,* community worker from a migrant background working in a child and family support community centre]*.

HCPs also sensed general distrust towards HCPs and services among migrant women, as some women tended to feel threatened or fear misunderstanding or child protection involvement. According to participants, several strategies facilitated trust building: genuine curiosity and respect for cultural differences, sufficient time, continuity of care, and familiar settings such as community centres or women’s homes.

#### Screening in its context

##### Ambivalence about screening

Contrasting perspectives regarding the importance and acceptability of screening was noted within and between participants. While some valued screening for its alerting function, others questioned its effectiveness or relevance, particularly for families in vulnerable situations facing more pressing challenges. Also, contrasting practices occurred, with each HCP having their own approach to screening, using different instruments, or not using any instrument at all. Structured questionnaires were appreciated by some (e.g. for initiating conversations and educational purposes) while others found them artificial or prone to socially desirable responses. Informal approaches (such as observing interactions or asking indirect questions) were preferred by those HCPs relying more on intuition. Concerns were raised about cultural sensitivity and screening being a one-time ‘checklist’ without follow-up. Some also reported lacking knowledge of and access to screening tools and called for training.

*I think it is certainly interesting as a healthcare provider to have a few items of this in mind that you can question*,* can question specific- different domains*,* for example*,* though. But to really administer it in a standardised way yes*,* I have my doubts about the effectiveness of that. [FG2*,* GP working in a community health centre]*

Time, responsibility, and the setting of screening were also discussed. Early screening at intake was formulated as important but hindered by time constraints and a lack of a trusting relationship at early stages. HCPs advocated for screening at every encounter and a shared responsibility across HCPs, although this could lead to screening not being conducted at all. Preferences regarding who should screen varied, with some favouring GPs and others preferring midwives or physiotherapists, noting they often have more time and room for an informal discussion of one’s mental state. Community workers were also suggested as particularly suitable because of their accessibility; however, one community worker cautioned that such involvement might feel too frightening to some. Additionally, the setting was influential, with rushed or formal environments hindering openness, and home visits or community-based settings facilitating disclosure.

##### Organisational and financial factors

Several broader organisational and financial structures were seen as barriers, particularly the fee-for-performance structure in the Belgian healthcare system, discouraging time-intensive tasks such as screening, interdisciplinary collaboration, or the use of interpreters. Although some participants felt uncomfortable asking for it, they acknowledged the facilitating potential of additional funding or flat-rate payment models.

*I think if you really want to make a change*,* there’s already so much*,* time pressure and then adding that on top of it. But if you know*,* ‘okay if I do it*,* I’ll actually be paid for it too’. I think that helps take the step to actually do it and keep doing it. And maybe it’s bad to say that we need money for it. But you already have to do so much in that one hour. [FG4*,* midwife working in a community health centre]*

Data-sharing systems also impacted care delivery. While some HCPs advocated for direct file access to improve efficiency and continuity of care, others expressed privacy concerns. Professional secrecy also hindered communication between HCPs, though integrating screening into medical records facilitated screening normalisation and visibility. Additionally, multidisciplinary collaboration was seen as beneficial, allowing validation of clinical impressions and task sharing. On the contrary, collaboration with external HCPs, e.g. GPs, midwives or child health services, was experienced as insufficient, with HCPs calling for case discussions and more regular contact.

#### Accessible referral

Following a positive screening, participants typically explored the mother’s needs and empowered her before considering referral. Frustration arose when referrals lacked follow-up, prompting them to intervene, leading to increased workload and unclear boundaries. While GPs were seen as central in the referral process, destinations also included child health services, primary care psychologists, hospital social services, specialised psychiatric services, and peer support groups. Despite this wide offer, HCPs highlighted long waiting lists, high costs, and a lack of accessible and culturally and linguistically appropriate care as barriers. These barriers were especially pronounced for specialised care, where migrant mothers faced logistical challenges and, at times, discrimination due to language needs. These barriers were overcome by direct referral to trusted professionals in familiar settings without waiting lists, and when not possible, guidance to services by familiar HCPs.

*We really have yes people who really need very intensive guidance of which they say, ‘yes*,* look the knowledge of Dutch is insufficient to be able to work with you’. Point. And that is really*,* yes*,* yes*,* and that is sometimes really frustrating. [FG5*,* GP working in a community health centre]*

#### PMH in its proper context

##### Stigma, taboo, PMH literacy and understanding of PMH

Stigma and taboo surrounding PMH were identified as major barriers towards disclosure. HCPs noted that many mothers, migrant and non-migrant, tended to mask or normalise their complaints. Particularly among migrant communities, HCPs noted how PMH was often taboo, perceived as ‘white people issues’ or signs of being crazy, with coping mechanisms rooted in faith. Moreover, some participants expressed discomfort discussing mental health.

*And what I also find really hard is the mental side of things*,* like when they don’t want to admit*,* that things are tough. When they say*,* ‘I’m strong*,* I can handle it’*,* that’s hard too. I’m like, ‘yes*,* you can do that*,* I know that*,* but it’s hard’. And maybe that is cultural. […] They often put on a mask and act like everything’s fine*,* and I just sit down with them and let it come out naturally. [FG3*,* community worker from a migrant background working in a child and family support community centre]*

Limited mental health literacy further hindered screening. HCPs noted that some migrant mothers struggled to understand screening questions or to express themselves or were unfamiliar with mental health care, requiring additional time and education during the consultation. Despite this, some HCPs appreciated mothers’ unique understanding of PMH and favoured asking about mental health views in their country of origin. Consequently, HCPs called for training on culture-sensitive communication and PMH screening, suggesting its inclusion in standard curricula.

*I think there’s so much wisdom in that [women’s own understanding of PMH]. I’ve already learned so much from it. And then I’m like, ‘wow*,* we can really learn a lot from this’. [FG5*,* primary care psychologist working in a community mental health centre]*

To address differing understandings, participants identified several facilitators. These included normalising emotional difficulties and help-seeking, offering educational sessions and launching broader awareness campaigns. Respectful dialogue and self-reflection were seen as key to fostering a shared understanding of PMH. Moreover, peer testimonies, shared cultural backgrounds, and acknowledgement of cultural differences were suggested to help bridge gaps in perception.

##### The involvement of ‘the village’

Involvement of the village, i.e. women’s partner, family, and broader support system, was described as both a potential facilitator and barrier to screening and referral. While their presence could foster wellbeing and offer opportunities for education around PMH and parenting, it could also hinder openness, especially when partners acted as interpreters, raising concerns about the accuracy and completeness of communication.

*Uhm. It’s often the partner who translates. In many families*,* it’s the partner who comes along*,* usually the man. […] So they end up translating*,* and you get the info through the partner. Which means you’re dealing with a middle person. And that’s not always super objective. So yeah*,* that makes things more complicated too. [FG4*,* midwife working in a community health centre]*

Recognising the partner’s role was seen as key, with requests for specific tools to facilitate partner communication. Beyond the partner, insight into the woman’s broader system was valued. Observing mother-child interactions and asking about their relationship with their own mother also provided valuable insights for them.

### Member check

Five participants completed the member check (RR = 25%) (Additional file 3). Responses ranged from neutral (*n* = 1) to positive (*n* = 2) and very positive (*n* = 2) regarding the relevance of each subtheme to their experiences and needs. No missing elements were reported, and all agreed on the formulation of the themes.

## Discussion

This study explored HCPs’ experiences and needs regarding PMH screening and referral among (expectant) mothers from migrant backgrounds. Our findings demonstrate that contrasting attitudes toward screening are shaped by cultural and linguistic barriers, levels of (dis)trust, organisational and financial factors, stigma, PMH literacy, and the complex interactions between healthcare professionals, mothers, and their networks, resulting in inconsistency in practice. HCPs also reported challenges in referring women to specialised care, and expressed the need for accessible, culturally and linguistically appropriate services. Together, these findings underscore the complex and multifaceted nature of HCPs’ experiences, providing a foundation for the following discussion of how these factors interact to shape PMH care for migrant mothers.

Our findings underscore the complexity of interactions influenced by cultural and language barriers. To bridge cultural barriers, cultural support workers were sometimes used to navigate cultural differences. Although their value in refugee mental health care has been demonstrated [42], their role in primary care and community-based settings remains unexplored. Moreover, language barriers often hinder interactions between HCPs and expectant mothers. Language support services, such as interpreters, were commonly used and generally facilitated communication [26, 27]. Nonetheless, their use was sometimes limited by practical and attitudinal barriers, with some HCPs perceiving them as unnecessary. Video-mediated interpreting may help overcome practical barriers [43], however, research shows that systemic barriers often persist [44]. This highlights the need for coordinating interventions addressing practical, attitudinal and systemic barriers [44].

Our results further underscore the importance of a trusting relationship between HCPs and their patients for disclosure and (acceptance of) referral, aligning with prior research [27, 45–48]. To facilitate this trust building, several factors were identified, in line with previous literature: sufficient time [48, 49], continuity of HCP [45, 48, 50], curiosity and respect for cultural differences [29]. In addition to the factors and drawing on research from the field of psychotherapy, we additionally suggest that the facilitating role of operationalising epistemic trust in screening and referral practices could further be explored [51]. This approach has shown potential in enhancing effective psychotherapy and might facilitate disclosure of mental health problems in non-therapeutic settings [51].

Moreover, some women seemed to prefer culturally similar HCPs, occasionally refusing referrals to unfamiliar HCPs. Participants from migrant backgrounds themselves felt that these choices sometimes placed excessive demands on them, leading to role strain, balancing clinical work with informal advocacy or interpreting. To protect their well-being, HCPs from these culturally diverse communities should receive sufficient support, especially as more representation in the workforce is needed to increase cultural competency and trust in services [24].

Additionally, our results illustrate diverse HCPs’ perceptions about screening practices. Although screening was conceived as an important practice, HCPs also questioned it in terms of priority and effectiveness. This result contrasts with previous studies, reporting a general acceptability of PMH screening by HCPs [52, 53], and international guidelines, recommending universal screening for all perinatal women [54–56]. This finding also stresses that changes in attitudes toward screening are necessary to align practice with best-practice recommendations, which could be achieved through training that addresses HCPs’ attitudes, knowledge, and skills [57]. Ambivalence was also expressed towards structured instruments, which were perceived as both useful and overly mechanical, particularly when applied in a checklist-like manner. This concern was echoed by perinatal women, who described the tick-box feature as a barrier to screening [58]. During the focus groups, the cultural sensitivity of translated instruments was questioned, reflecting previous studies questioning the translations of the Edinburgh Postnatal Depression Scale (EPDS) or General Health Questionnaire (GHQ) [29, 45, 59–61]. These concerns were reflected in some HCPs’ preference for an informal, intuitive approach, described before as tacit knowledge, described as experience-based, pragmatic and context-specific, difficult to articulate, and shared through dialogue and experience [29, 62]. While tacit knowledge plays a crucial role in supporting clinical judgment, evidence also emphasises the importance of routine standardised screening in increasing referrals and service use, thereby promoting more equitable identification of PMH concerns [12, 13]. Given the complementary strengths of both approaches, we argue that tacit knowledge should not be viewed as separate from standardised protocols, but rather integrated into them, ideally through co-design with clinicians and service users [63].

Regarding referral practices, our results showed systemic barriers, illustrating the importance of accessibility and person-centred care. HCPs were frustrated by poor follow-up by GPs, echoing the experiences reported by mothers [24]. HCPs felt sometimes forced to intervene themselves in order that their mothers would receive the necessary care. HCPs often act as “navigators” for migrant women in the healthcare system, and see it as an important part of their work [27]. However, some HCPs struggled with the additional workload and unclear professional boundaries that this role entails [64]. Consistent with previous research, referral barriers further included long waiting lists [64–66], high costs [66], and a lack of culturally appropriate services [65]. This highlights the urgent need for policy changes to address and overcome these systemic barriers.

Furthermore, our findings highlight discomfort among HCPs discussing PMH, possibly stemming from limited knowledge or confidence [24, 27], or from perceiving mental health as taboo or from stigmatising attitudes, expressed as fear of causing offence [67, 68]. These findings again underscore the importance of training that enhances HCPs’ PMH knowledge, builds confidence, and addresses negative or avoidance-based attitudes towards PMH [68]. Additionally, HCPs stated that low PMH literacy among mothers was often felt as a barrier, limiting the accuracy of screening [24, 27, 29, 45, 69]. This feeling further highlights the value of educational initiatives for migrant women and their network aimed at improving the understanding of PMH within diverse communities.

Finally, HCPs stated that partners and families seem to both hinder and support PMH disclosure, consistent with prior research [24, 26]. Women who feared disapproval from their families were less likely to answer screening questions honestly or to accept referral compared with those who felt supported [45, 70]. Although HCPs appreciate partner involvement, they raised concerns about gatekeeping or interpretation by partners. Recent literature reports similar findings, suggesting that interpreting by partners can lead to skipped screening, pleading for confidential, separate screening for women and their partners, as well as the use of professional interpreters [71]. This approach can also be used for awareness raising among partners, though its feasibility needs further study.

Building on our main findings, several implications arise for clinical practice and future research. First, for clinical practice, HCPs should focus on trust building through respectful, curious engagement, especially towards cultural differences. Furthermore, we recommend training on PMH knowledge and screening, referral and cultural and language support, to further strengthen HCPs’ attitudes, knowledge and confidence. At a policy level, efforts should focus on systemic barriers to screening and referral, with a specific focus on accessible, affordable and culturally appropriate care. Because of the need for additional training, further research should focus on educational intervention strategies for HCPs, which have been shown to be feasible [72]. Future research should focus on refining these strategies and evaluating their effectiveness and cost-efficiency. Building on participants’ calls for additional support, further research should also focus on the development of reliable and accessible tools to support PMH discussions, including with family. Finally, as our findings only shed light on HCPs’ perspectives, future research should explore how these processes are experienced and perceived by perinatal women from migrant backgrounds themselves to further refine processes in a culturally sensitive way.

### Methodological considerations

Some limitations should be considered, although several were mitigated, and some contributed to the depth of the findings. First, due to organisational constraints, some focus groups were smaller and/or more homogenous than initially planned, which might have influenced group dynamics and limited diverse perspectives [37]. Therefore, social desirability may have led some participants to withhold or moderate their opinions. However, smaller groups might also have created safer environments for open discussions [37]. Second, the sample was limited to Dutch-speaking participants, reflecting the linguistic reality of Flemish healthcare, where Dutch is the primary language of communication. This may have limited the participation of non-native Dutch-speaking HCPs, who could offer distinct insights into cultural and language-related challenges in care delivery. Nevertheless, we were able to include five non-native Dutch-speaking participants, which partially mitigates this limitation. Third, some participants lacked formal screening experience, occasionally causing misunderstandings, but also enriching the discussion and highlighting the challenges and opportunities for effective implementation. Lastly, only five of twenty participants (RR = 25%) responded to the member check survey. The reason for non-responding is unknown, and because the survey was anonymous, we were unable to assess whether nonresponse was random. Nevertheless, the low response rate may be explained by limited time, and the perception that the survey offered little added value after participants had already shared their views extensively in the focus groups.

## Conclusions

To promote equitable PMH screening and referral practices, this study explored HCPs’ experiences and needs regarding these clinical practices among migrant populations. Central were the impact of cultural and language barriers, and a trusting relationship as a crucial condition for effective care. Furthermore, HCPs expressed a certain ambivalence towards screening and its implementation and highlighted the impact of systemic factors on their ability to screen. Moreover, HCPs called for accessible, culturally and linguistically appropriate referral, preferably to a familiar professional or setting, or guided by a trusted individual. Finally, HCPs highlighted stigma, taboo, limited PMH literacy and understanding of PMH among perinatal women and themselves as barriers to disclosure and emphasised the dual role of women’s partner and family. Based on these findings, researchers and policymakers can further design and implement screening and referral protocols, ideally in collaboration with HCPs and perinatal women, to achieve equitable care practices for all.

## Supplementary Information

Below is the link to the electronic supplementary material.

Supplementary Material 1

Supplementary Material 2

Supplementary Material 3

## Data Availability

The data that support the findings of this study are not openly available due to reasons of sensitivity and are available from the corresponding author upon reasonable request. Data are located in controlled access data storage at KU Leuven.

## Abbreviations

GP: General practitioner
HCP: Healthcare provider
PMH: Perinatal mental health
