# Supplementary material for: Equitable and culturally sensitive perinatal mental health screening and referral for all: experiences and needs from primary care and community-based healthcare providers
Source: BMC Health Serv Res. 2026 Mar 11;26:468. doi: 10.1186/s12913-026-14346-z (PMC13049847; doi:10.1186/s12913-026-14346-z)
Supplement: Supplementary file 1 — Supplementary Material 1 [file 12913_2026_14346_MOESM1_ESM.docx]

# Additional file 1

## Topic guide

At the start of each focus group, the moderator and observer introduced themselves and informed the participants about the study including the broader research project, the study’s aims and the topic of the discussion. The moderator also provided the participants with a set of guidelines to ensure the focus group discussion proceeded smoothly. Next, participants were asked to introduce themselves, by giving their name, age, professional background and experience, and by discussing whether they had a migrant background themselves. Afterwards, the main discussion started.

The following pre-defined topics were discussed:

1. Experience with working with mothers from migrant backgrounds in general;
2. Experience with talking about mental health with mothers from migrant backgrounds ;
3. Experience with PMHP screening among with mothers from migrant backgrounds: current practice, barriers, facilitators;
4. Perspectives on future screening: needs and preferences regarding approach, setting, and perspectives on strategies to facilitate implementation in clinical practice.

The following questions were asked:

1. How do you experience working with (expectant) mothers with migrant background?
   - What is going well?
   - What is challenging?
2. How do you experience talking about mental health with (expectant) mothers migrant background?
   - What is going well?
   - What is challenging?
3. How do you detect perinatal mental health problems in (expectant) mothers with migrant background?
   - Do you use any screening programs or tools? How does this work?
   - When do you do this? What do you think is the most suitable time to screen?
4. How and when does referral happen?
   - What is challenging about that?
   - Suppose a woman indicates that things are not going well, or you feel that something is not right, what do you do?
5. Do you think there is an ideal HCP or an ideal moment for screening?
6. What do you think could help to improve the screening for mental health problems among (expectant) mothers with migrant background?
   - In terms of cultural sensitivity?
   - Do you think of any other factors that could make the process easier?
   - Which promoting factors do you think are the most important?
   - What things should not change?

During that fifth and last focus group discussion, an additional question was asked based on preliminary analyses of the first few transcripts.

1. Our previous focus groups showed that care providers rely heavily on their gut feeling and seem to have little need for a formal/standardized tool.
   - Is this something you recognize yourself in?
   - How important is this screening to you?

To conclude, participants were asked what they found the most important thing they discussed during the focus group. Finally, participants were extensively thanked for their participation, and it was reiterated that their data would be handled confidentially. It was also emphasized that they could always contact the researchers in case of any questions or comments.
