# Supplementary material for: Equitable and culturally sensitive perinatal mental health screening and referral for all: experiences and needs from primary care and community-based healthcare providers
Source: BMC Health Serv Res. 2026 Mar 11;26:468. doi: 10.1186/s12913-026-14346-z (PMC13049847; doi:10.1186/s12913-026-14346-z)
Supplement: Supplementary file 2 — Supplementary Material 2 [file 12913_2026_14346_MOESM2_ESM.docx]

# Additional file 2

## Member check: survey

### Feedback focus groups on culturally sensitive screening of perinatal mental health

A while ago, you participated in one of our five focus groups on culturally sensitive screening and referral in perinatal mental health. Once again, thank you very much for your valuable contribution!

We analysed these five sessions, with input from 20 participants, using *framework analysis*, a method for processing qualitative data such as focus group discussions. This analysis revealed seven sub-themes, grouped under four main themes.

We’ve summarised these themes in a clear **infographic,** click here to view it.

To find out whether these themes also resonate with you, we kindly ask you to complete for a short **questionnaire**. Your feedback helps us interpret and refine the results accurately.

- Duration: max. 5 to 10 minutes
- Deadline: Sunday, 10 August 2025, 11:55 PM
- Anonymous: yes

Do you have any questions or comments? Do not hesitate to contact me at floor.puttemans@kuleuven.be.
**Thank you in advance for your additional contribution!**

Section 1

### General impression

1. To what extent do the themes reflect your experiences and needs in general?*

| Not at all | Not really | Neutral | Somewhat | Very much |
| --- | --- | --- | --- | --- |
| ○ | ○ | ○ | ○ | ○ |

1. Would you like to elaborate on your answer? Please explain.

[Open field]

Section 2

### Theme *Stigma, taboo, literacy and understanding of perinatal mental health (PMH)*

- **Stigma and taboo** hinder openness among both migrant and non-migrant women, and healthcare providers. **Limited mental health literacy and differing understandings of PMH**pose challenges.
- **Acknowledging** stigma, taboo and differences in understanding creates connection, but knowledge in this area is lacking.
- **Education, training and awareness-raising** can help.

1. To what extent do you recognize the theme of *Stigma, taboo, literacy and understanding of* perinatal mental health (PMH) based on your experiences and needs?*

| Not at all | Not really | Neutral | Somewhat | Very much |
| --- | --- | --- | --- | --- |
| ○ | ○ | ○ | ○ | ○ |

1. Would you like to add or nuance anything regarding this theme?

[Open field]

Section 3

### Theme *Involvement of 'the village'*

- Involvement of **partner and family** influences screening and referral.
- Cooperation with **other healthcare providers** plays an important role.
- The impact can **be positive or negative**, depending on the context.

1. To what extent do you recognize the theme *of Involvement of 'the village'* based on your experiences and needs?*

| Not at all | Not really | Neutral | Somewhat | Very much |
| --- | --- | --- | --- | --- |
| ○ | ○ | ○ | ○ | ○ |

1. Would you like to add or nuance anything regarding this theme?

[Open field]

Section 4

### Theme *Cultural and language barriers*

- **Differences in culture and language** complicate communication and care provision.
- **Tools, interpreters and culturally sensitive support workers**are used, but concerns arise about quality, costs and availability, and insufficient provision and knowledge.
- **Training and accessible and reliable tools** are urgently needed.

1. To what extent do you recognize the theme *of Cultural and Language Barriers* based on your experiences and needs?*

| Not at all | Not really | Neutral | Somewhat | Very much |
| --- | --- | --- | --- | --- |
| ○ | ○ | ○ | ○ | ○ |

1. Do you want to add or nuance anything regarding this theme?

[Open field]

Section 5

### Theme *Ambivalence around screening*

- **Opinions** on the importance, format and implementation of screening **vary** among healthcare providers.
- **Time, space and atmosphere** influence the success of screening.
- Healthcare providers are concerned about **cultural sensitivity**, but screening also offers **educational opportunities for migrant women**.

1. To what extent do you recognize the theme *of Ambivalence around screening* based on your experiences and needs?*

| Not at all | Not really | Neutral | Somewhat | Very much |
| --- | --- | --- | --- | --- |
| ○ | ○ | ○ | ○ | ○ |

1. Do you want to add or nuance anything regarding this theme?

[Open field]

Section 6

### Theme *Social factors*

- **‘Fee-for-performance’ structures and limited access to medical records** hinder screening.
- Strict regulations on **professional secrecy** complicate collaboration between healthcare providers.
- **Additional financial incentives** could facilitate screening and language support.

1. To what extent do you recognize the theme *of Societal factors* based on your experiences and needs?*

| Not at all | Not really | Neutral | Somewhat | Very much |
| --- | --- | --- | --- | --- |
| ○ | ○ | ○ | ○ | ○ |

1. Do you want to add or nuance anything regarding this theme?

[Open field]

Section 7

### Theme *Relationship of trust*

- Trust is **crucial** for effective care.
- **General distrust** towards healthcare providers and **language barriers** hinder the development of a trusting relationship.
- **Time, continuity, working in the home context and acknowledging cultural differences** help build trust.

1. To what extent do you recognize the theme *of Relationship of trust* based on your experiences and needs?*

| Not at all | Not really | Neutral | Somewhat | Very much |
| --- | --- | --- | --- | --- |
| ○ | ○ | ○ | ○ | ○ |

1. Do you want to add or nuance anything regarding this theme?

[Open field]

Section 8

### Theme *Accessible referral*

- **Healthcare providers often provide initial support themselves** before referring. **Referrals are made to a range of healthcare providers and services** within primary care, specialized care and community organizations.
- **Referral processes are hindered** by waiting lists and limited provision, especially in specialized care.
- **A familiar context and guidance** by a truster person make referrals more accessible for migrant women.

1. To what extent do you recognize the theme *of Accessible referral* based on your experiences and needs?*

| Not at all | Not really | Neutral | Somewhat | Very much |
| --- | --- | --- | --- | --- |
| ○ | ○ | ○ | ○ | ○ |

1. Do you want to add or nuance anything regarding this theme?

[Open field]

Section 9

### Reflections and suggestions

1. Is there anything you feel is missing? Please explain.*

[Open field]

1. Are the themes clearly and understandably formulated?*

- Yes
- No

**IF 18. = "No":**

1. What would you formulate differently?

[Open field]

Thank you

Thank you for your contribution! Your feedback helps us to correctly interpret and refine the results. We would be happy to share the final results with you.

*Required questions
