# Supplementary material for: Equitable and culturally sensitive perinatal mental health screening and referral for all: experiences and needs from primary care and community-based healthcare providers
Source: BMC Health Serv Res. 2026 Mar 11;26:468. doi: 10.1186/s12913-026-14346-z (PMC13049847; doi:10.1186/s12913-026-14346-z)
Supplement: Supplementary file 3 — Supplementary Material 3 [file 12913_2026_14346_MOESM3_ESM.docx]

# Additional file 3

## Member check: results

| Topics | Likert scale |
| --- | --- |
| Recognition of subthemes in general | 1x neutral,  2x somewhat,  2x very much |
| Recognition of *Stigma, taboo, literacy and understanding of perinatal mental health* | 3x somewhat,  2x very much |
| Recognition of *Involvement of ‘the village’* | 1x somewhat,  4x very much |
| Recognition of *Cultural and language barriers* | 4x somewhat,  1x very much |
| Recognition of *Ambivalence about screening* | 1x neutral,  4x somewhat |
| Recognition of *Societal factors* | 2x neutral,  1x somewhat,  2x very much |
| Recognition of *Relationship of trust* | 3x somewhat,  2x very much |
| Recognition of *Accessible referral* | 2x somewhat,  3x very much |
| Missing elements | N/A |
| Understandability | 5x yes |

Abbreviations: N/A, No Answer
